# Hepatic Vitamin A Concentrations and Association with Infectious Causes of Child Death

**DOI:** 10.1016/j.jpeds.2023.113816

**Published:** 2024-02

**Authors:** Priya M. Gupta, Zachary J. Madewell, Bryan M. Gannon, Michael Grahn, Victor Akelo, Dickens Onyango, Sana Mahtab, Shabir A. Madhi, Judith Giri, Dianna M. Blau, Usha Ramakrishnan, Aryeh D. Stein, Cynthia G. Whitney, Melissa F. Young, Sherry A. Tanumihardjo, Parminder S. Suchdev

**Affiliations:** 1 Hubert Department of Global Health, Rollins School of Public Health, Emory University, Atlanta, GA; 2 Global Health Center, Centers for Disease Control and Prevention, Atlanta, GA; 3 Department of Nutritional Sciences, University of Wisconsin-Madison, Madison, WI; 4 US Centers for Disease Control and Prevention-Kenya, Kisumu, Kenya; 5 County Department of Health, Kisumu, Kenya; 6 South African Medical Research Council Vaccines and Infectious Diseases Analytics Research Unit, University of the Witwatersrand, Johannesburg, South Africa; 7 Department of Pediatrics, Emory University, Atlanta, GA

**Keywords:** malnutrition, pediatric infections, under-5-mortality, vitamin A

## Abstract

**Objectives:**

To assess postmortem vitamin A (VA) concentrations in children under 5 years of age and evaluate the association between VA deficiency (VAD) and infectious causes of death (CoD).

**Study design:**

In this cross-sectional study from the Child Health and Mortality Prevention Surveillance (CHAMPS) Network, liver biopsies collected within 72 hours of death were analyzed from 405 stillbirths and children under 5 years in Kenya and South Africa. Total liver VA (TLVA) concentrations were quantified using ultra-performance liquid chromatography, and cutoffs of ≤0.1 μmol/g, >0.1 to <0.7 μmol/g, ≥0.7 to <1.0 μmol/g, and ≥1.0 μmol/g were used to define VAD, adequate VA status, high VA, and hypervitaminosis A, respectively. CoD were determined by expert panel review.

**Results:**

Among 366 liver samples with viable extraction, pooled prevalences of VAD, adequacy, high VA, and hypervitaminosis were 34.2%, 51.1%, 6.0%, and 8.7%, respectively. VAD was more common among neonates compared with stillbirths, infants, or children, and among those with low birthweight (LBW), underweight, or stunting (*P* < .05). When adjusting for site, age, and sex, there was no significant association of VAD with increased infectious CoD (OR 1.9, 95% confidence interval [CI] 0.9, 3.8, *P* = .073). In stratified analyses, VA deficient boys, but not girls, had an increased risk of infectious CoD (OR 3.4, 95% CI 1.3, 10.3, *P* = .013).

**Conclusions:**

Definitive postmortem assessment of VA status identified both VAD and VA excess among children under 5 years of age in Kenya and South Africa. VAD in boys was associated with increased risk of infectious mortality. Our findings may inform a transition from universal VA supplementation (VAS) to targeted strategies in certain countries.

Vitamin A (VA) is a fat-soluble vitamin that plays a critical role in immune function, embryonic development, cell differentiation, metabolism, and vision.^1^ VA deficiency (VAD) contributes substantially to disease burden, specifically in Africa and South Asia, with the most severe effects noted during periods of rapid growth and development, such as pregnancy and the first 5 years of life.^2^

The World Health Organization (WHO) estimates that 19 million pregnant women and 190 million preschool age children (PSC, 6-59 months) have VAD.^3^ VAD is the leading cause of preventable blindness,^1^^,^^4^^,^^5^ and increases childhood morbidity and mortality due to infections such as measles, diarrhea, and malaria.^6^, ^7^, ^8^, ^9^, ^10^ VA promotes and regulates both innate and adaptive immune responses and therefore is vital in strengthening immune function as well as inducing the appropriate immune response against specific infectious diseases.^11^

VA supplementation (VAS) in early life is a well-established, cost-effective strategy to prevent under-5-year-old mortality (U5M). More specifically, VAS of children is associated with a 12%-24% reduction in all-cause U5M.^5^ Community and clinical trials have demonstrated that VA prophylaxis alleviates the effects of measles, diarrhea, vision problems, ear infections, and subsequent hearing loss. WHO recommends universal VAS with preformed VA at 100 000 IU dose to infants 6-11 months of age and 200 000 IU every 4-6 months to children 12-59 months in settings where prevalence of VAD exceeds 20% or the prevalence of night blindness exceeds 1%.^3^ Evidence-based strategies to combat VAD also include early initiation of breastfeeding, timely introduction of VA-rich complementary foods, biofortification, food fortification, and emphasis on infectious disease reduction.

Total liver VA concentration (TLVAC) is considered the reference standard for assessing the continuum of VA status from deficiency through excess.^1^^,^^12^^,^^13^ However, direct measurement of TLVAC requires liver biopsy, which typically is not feasible in living populations. While serum retinol and retinol-binding protein (RBP) are commonly used for VA assessment in population-based studies, these biomarkers do not reflect VA stores accurately, nor can they be used to classify individuals across the VA status continuum, from deficiency through excess.^1^^,^^4^^,^^14^, ^15^, ^16^, ^17^, ^18^

Eradication of VAD has been the focus of many public health interventions; however, there is no clear guidance on when to scale back universal VAS and whether VAS should continue in countries with multiple overlapping VA interventions.^18^

To address these research and programmatic knowledge gaps, we evaluated postmortem liver specimens along with detailed data on cause of death from the Child Health and Mortality Prevention Surveillance (CHAMPS) network to examine definitive VA status. We also sought to quantify the association between VAD and infectious causes of death (CoD) to inform universal VAS strategies.

## Methods

### Data Source

Details on the CHAMPS approach, sample selection, eligibility criteria, site characteristics, and specimen and data collection methods have been published in detail elsewhere.^19^, ^20^, ^21^, ^22^, ^23^, ^24^, ^25^, ^26^, ^27^, ^28^ In brief, CHAMPS collects standardized, population-based, surveillance data from sites in Africa and Southern Asia with high child mortality to understand and track preventable CoD. These data include demographic characteristics, extensive postmortem diagnostic results, and data from abstracting clinical medical records. Postmortem tissue samples are secured using minimally invasive tissue sampling (MITS).^20^^,^^21^^,^^25^^,^^27^^,^^29^ Data collection procedures were approved by ethics committees in each CHAMPS site as well as Emory University (Emory IRB#: 00 091 706). Consent for MITS, verbal autopsy, and clinical data abstraction was obtained from parents or guardians. CHAMPS ethical protocols are described in greater detail on the study website (https://champshealth.org/protocols/). This study followed the Strengthening the Reporting of Observational Studies in Epidemiology reporting guideline.

### Sample Selection and Exclusions

Liver samples (mean ± SD mass: 19 ± 8 mg) were procured via needle biopsy from 405 stillbirths and cadavers under 5 years within 72 hours of death (median of 19 hours) using standard procedures.^27^ Multiple biopsies were collected from needle puncture in the mid-axillary line, in 1 of the three last intercostal spaces with some tissue frozen as quickly as possible and remaining samples fixed in 10% neutral buffered formalin for histological evaluation. All samples collected between May 2017 and December 2019 from the CHAMPS Kenyan and South African sites and deposited in the CHAMPS central biorepository were included. Catchment areas in Kenya included Siaya (rural) and Kisumu (urban) and in South Africa included Soweto (urban). VAD (based on serum retinol) is high in Kenya and moderate in South Africa.^30^ However, both countries have overlapping VAS and fortification programs that may increase VA status in subsets of the population.^31^, ^32^, ^33^

### Exposure: Liver VA Concentrations

Liver samples were weighed to the nearest 0.001 mg on a Sartorius CP2P scale (range 1-46 mg), extracted with dichloromethane,^34^ and TLVACs (retinol and retinyl esters) were quantified using a published ultra-performance liquid chromatographic gradient and calibrated against a purified standard (Sigma)^35^ C23-β-apo-carotenol was used as an internal standard to measure and account for extraction efficiency; samples with extraction efficiencies <10% were excluded. The detection limit of the was 0.001 μmol/g for VA esters. Cutoffs for TLVAC of ≤0.1 μmol/g, >0.1 to <0.7 μmol/g, ≥0.7 to <1.0 μmol/g, and ≥1.0 μmol/g were used to define VAD, adequate VA status, high VA, and hypervitaminosis A, respectively.^1^^,^^36^

### Outcome: Infectious CoD

The Determination of CoD (DeCoDe) is a process to evaluate multiple CoD and decrease subjectivity, through a standardized approach of applying clinical and laboratory findings to diagnose the conditions contributing to death based on completeness and specificity of data. A complete description of the DeCoDe process has been published elsewhere.^19^ Briefly, stillbirths and deaths in children <5 years are identified in site catchment areas; following parental consent, MITS are collected and tissues examined using microbiology and pathology techniques. CHAMPS teams also review clinical records and interview parents using a standard verbal autopsy form. All available data for each death are then reviewed by site-specific DeCoDe panels that consist of subject matter experts including pediatricians, obstetricians, epidemiologists, pathologists, and microbiologists. The panels review available case data and determine the chain of events (immediate, underlying, comorbid causes) leading to death using WHO's International Classification of Diseases, 10th Revision and WHO application of International Classification of Diseases, 10th Revision deaths during the perinatal period.^37^^,^^38^ Immediate, underlying, and morbid CoD will subsequently be referred to as those within the causal chain.^19^

Our primary outcome of interest was all infectious CoDs within the causal chain defined as diarrheal diseases, congenital infections, human immunodeficiency virus, lower respiratory infection (LRI), malaria, measles, meningitis, encephalitis, neonatal sepsis, other infections, rabies, sepsis, syphilis, tuberculosis, or upper respiratory infections in the causal chain. Sub-analyses were performed to examine the association between VAD and infectious CoDs with an established relationship with VA, defined as deaths due to LRI s, sepsis, and diarrheal diseases.

### Statistical Analyses

We first developed frequency distributions to describe demographic characteristics stratified by age. Pearson chi-square tests and univariable models were used to examine relationships between covariates, exposure and outcomes when exploring potential confounders. Confounding covariates were also considered if sufficient evidence in existing literature warranted inclusion in the model.

Covariates evaluated as potential effect modifiers and confounders included: age (categorized as stillbirths and deaths in the first 24 hours, neonates [1 through 27 days], infants and children [28 days through 59 months]), site (Kenya, South Africa), and sex (male, female). We also examined locations of death (facility or community). Low birthweight (LBW) was defined as <2500 g. We also examined evidence of malnutrition based on anthropometry at death. Anthropometric measurements were collected during the MITS examination using standard equipment, including wooden height-length measuring boards (ShorrBoard, Weigh and Measure, LLC) and digital scales (Rice Lake Weighing Systems, Inc). Z-scores for anthropometric indices were produced using the WHO growth standards *anthro* R package.^39^ Rigor mortis did not prevent acquisition of accurate anthropometric measurements.^40^ We examined the individual effects of underweight, stunting, or wasting, as well as any malnutrition defined as the presence of either underweight, wasting, or stunting at death, using z-scores < −2 SD for weight-for-length (wasting), length-for age (stunting), or weight-for-age (underweight).

Ordinal logistic regression was used to quantify the relationship between the exposure VAD and all infectious CoD. The reference category for the exposure was adequate VA status. Models assessed for effect modification (*P* < .01), and if evidence of heterogeneity, stratified analyses were performed. Modeling was repeated with the outcome of select infectious CoD with an established relationship with VAD. Model significance was evaluated using *P* < .05. Odds ratios (OR) and 95% confidence intervals (95% CI) were reported. Model diagnostics, including Akaike Information Criteria, were considered when selecting final models. All analyses were completed in R statistical software (version 4.2.1, The R Foundation for Statistical Computing).^41^

## Results

**Figure 1:**
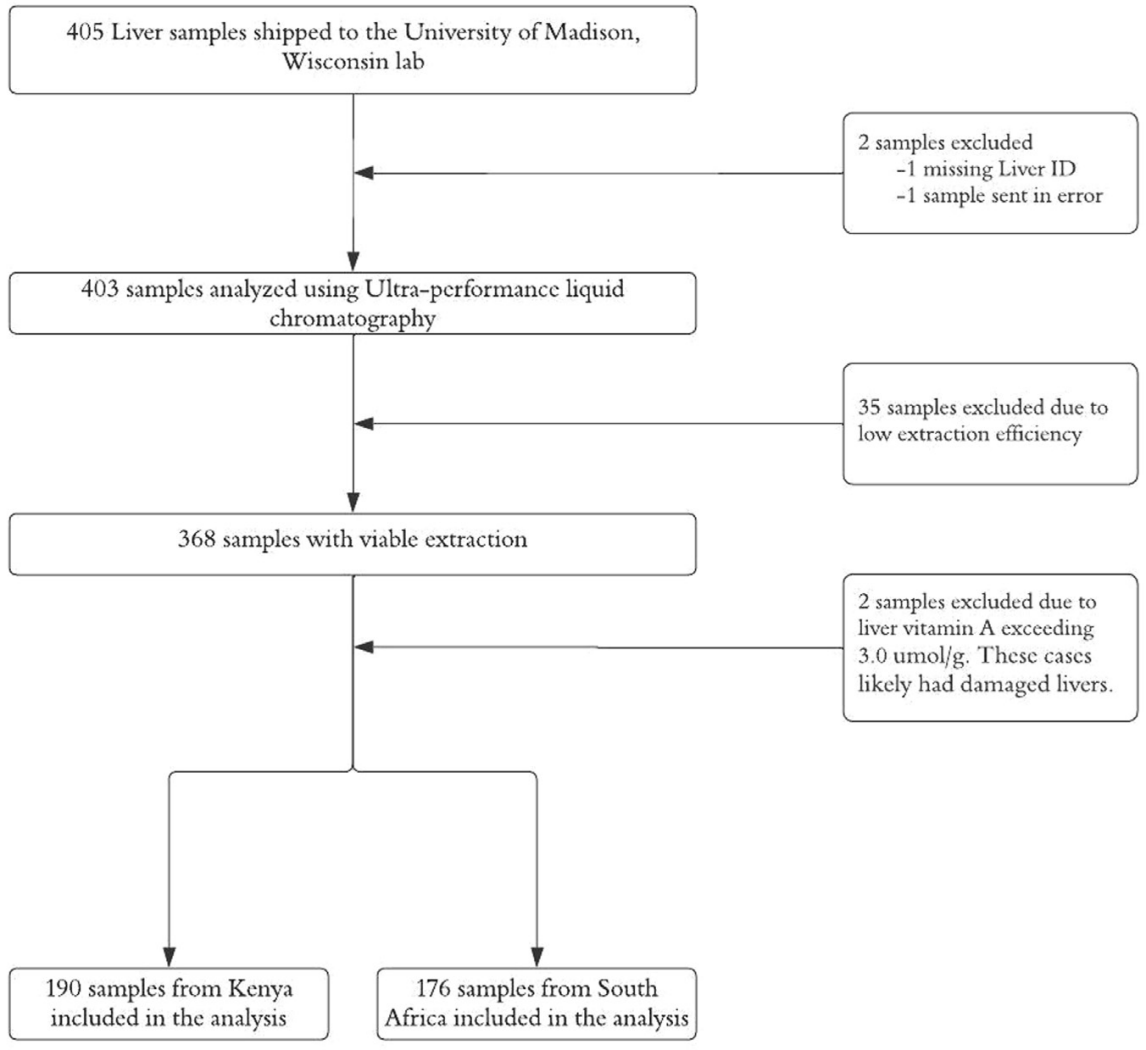
Study profile of children under 5 years with analyzed liver specimens from CHAMPS Kenya and South Africa Sites.

Of the 405 liver samples sent for analysis, 39 were excluded due to identification errors (n = 2), low or missing extraction efficiencies (n = 35), or TLVACs above 3.0 μmol/g (n = 2), which were assumed to be a result of damaged livers due to VA toxicity leaking retinyl esters into the serum.^42^ The majority of samples analyzed (n = 366, 90.3%) had good extraction efficiencies; 190 (51.9%) were from Kenya and 176 (48.1%) were from South Africa (Figure 1).

Nearly half enrolled children were infants and children (46.7%); 26.5% were stillbirths and deaths in the first 24 hours; and the remaining 26.8% were neonates (Table I). There was an even distribution of sex by age group. There were differences in age distributions by site and location of deaths with infants and children being most common in Kenya, and neonates most common in South Africa. Eight in 10 cases had evidence of malnutrition based on anthropometry at death, and the prevalence of underweight (79.4%) and stunting (69.9%) was significantly higher among neonates. Overall, nearly 2 in 3 deaths were due to infectious CoDs, and prevalence of infectious CoDs was highest among infants and children (90.1%), compared with 66.3% of neonates and only 2.9% of stillbirths or deaths in the first 24 hours (*P* < .01).

**Table I:** Demographic characteristics of children under 5 Years (n = 366) with analyzed liver specimens from CHAMPS Kenya and South Africa sites by age group

Characteristic	N	Overall	Age group n (%)∗			*P* value†
			Stillbirths or death in the first 24 hours	Neonates (1 to 27 days)	Infants and children (28 days to < 60 months)	
Overall	366		97 (26.5)	98 (26.8)	171 (46.7)	-
CHAMPS site, n (%)	366					**<.001**†
Kenya		190 (51.9%)	68 (70.1%)	14 (14.3%)	108 (63.2%)	
South Africa		176 (48.1%)	29 (29.9%)	84 (85.7%)	63 (36.8%)	
Sex, n (%)	366					.85†
Females		152 (41.5%)	39 (40.2%)	43 (43.9%)	70 (40.9%)	
Location of death, n (%)	366					**<.001**†
Community		65 (17.8%)	4 (4.1%)	4 (4.1%)	57 (33.3%)	
Facility		301 (82.2%)	93 (95.9%)	94 (95.9%)	114 (66.7%)	
Low Birthweight‡, n (%)	278‡	157 (56.5%)	53 (55.2%)	78 (82.1%)	26 (29.9%)	**<.001**†
Underweight‡, n (%)	361‡	232 (64.3%)	56 (58.3%)	77 (79.4%)	99 (58.9%)	**.001**†
Stunted‡, n (%)	341‡	168 (49.3%)	42 (45.7%)	58 (69.9%)	68 (41.0%)	**<.001**†
Wasted‡, n (%)	242‡	144 (59.5%)	30 (57.7%)	24 (75.0%)	90 (57.0%)	.16†
Any malnutrition‡, n (%)	358‡	288 (80.4%)	76 (79.2%)	91 (92.9%)	121 (73.8%)	**<.001**†
Infectious disease deaths‡, n (%)	366	226 (61.7%)	7 (7.2%)	65 (66.3%)	154 (90.1%)	**<.001**†
Lower respiratory infections, sepsis, or diarrheal diseases‡, n (%)	366	175 (47.8%)	5 (2.9%)	59 (33.7%)	111 (63.4%)	**<.001**†

Weight-for-age (WAZ), length/height-for-age (LAZ), and weight-for-length/height (WLZ) z-scores <2 SD was used to define underweight, stunted, and wasted, respectively. Sample decrease within the underweight category due to 3 cases missing weight, 1 case missing age in days, and 1 case with WAZ outside of defined limits. Sample decrease within the stunted category due to 20 cases missing z-score due to lengths under 45 cm and 5 cases with LAZ outside of defined limits (refer to methods section). Sample decrease within the wasting category due to 117 cases missing z-score due to lengths under 45 cm, 3 cases with missing weight, 3 cases outside of the defined upper limit, and 1 case with outside the measurement limits for raw length captured by the WHO-GS. Differences in sample size for wasting and stunting exist because LAZ is estimable based on length according to completed age and sex, whereas wasting is a measure of weight by length, details on the WHO-GS can be referenced elsewhere.^41^

Any malnutrition was defined WAZ, LAZ, or WLZ < -2 SD at death. Sample size drop in any malnutrition based on anthropometry at death due to 1 measurement outside of the WHO-GS bounds, and 7 cases with z-scores outside of the limits captured by the WHO-GS.

All Infectious diseases deaths included: diarrheal diseases, congenital infections, HIV, lower respiratory infections, malaria, measles, meningitis/encephalitis, neonatal sepsis/sepsis, other infections, rabies, syphilis, tuberculosis, or upper respiratory infections in the causal chain. Sub-analyses were conducted to investigate the association between vitamin A deficiency and select infectious causes of death with an established relationship with vitamin A. These conditions included: included: lower respiratory infections, neonatal sepsis/sepsis, and diarrheal diseases.

∗ n (%).

† Pearson's Chi-squared tests examining frequency distributions of demographic characteristics by age group.

‡ Birthweight was abstracted from maternal or child clinical records. For deaths and stillbirths in the first 24 hours, if clinical abstraction data was missing, birthweight was assumed to be the MITS weight. Low birthweight was defined as <2500 g. Eighty-seven cases missing clinical abstraction data from maternal and child clinical records; 1 stillbirth or death in the first 24 hours was missing both clinical abstraction weight and MITS weight.

Pooled prevalence of VAD, adequate VA, high VA, and hypervitaminosis was 34.2%, 51.1%, 6.0%, and 8.7%, respectively (Table II). The distribution of TLVACs differed significantly by age but not by site, sex, location of death, wasting, or infectious CoD. VAD was highest in neonates (39.8%) and lowest in infants and children (29.8%). Conversely, high or hypervitaminosis was highest in older children. Cases that were LBW, underweight, stunted, or had any evidence of malnutrition based on anthropometry at death had higher prevalence of VAD.

**Table II:** Vitamin A status by demographic characteristics of children under 5 years (n = 366) with analyzed liver specimens from CHAMPS Kenya and South Africa sites

Characteristic	N	Vitamin A Status∗				*P*-value†
		Deficient	Adequate	High	Hypervitaminotic	
Overall, N(%)	**366**	**125 (34.2%)**	**187 (51.1%)**	**22 (6.0%)**	**32 (8.7%)**	-
CHAMPS site, n (%)‡						
Kenya	190	60 (31.6%)	99 (52.1%)	14 (7.4%)	17 (8.9%)	.56†
South Africa	176	65 (36.9%)	88 (50.0%)	8 (4.5%)	15 (8.5%)	
Sex, n (%)						
Females	152	49 (32.2%)	80 (52.6%)	9 (5.9%)	14 (9.2%)	.93†
Age group, n (%)						
Stillbirths or Death in the first 24 hours	97	35 (36.1%)	49 (50.5%)	6 (6.2%)	7 (7.2%)	**.013**†
Neonates (1 to 27 days)	98	39 (39.8%)	55 (56.1%)	2 (2.0%)	2 (2.0%)	
Infants and Children (28 days to < 60 months)	171	51 (29.8%)	83 (48.5%)	14 (8.2%)	23 (13.5%)	
Location of death, n (%)						
Community	65	18 (27.7%)	32 (49.2%)	7 (10.8%)	8 (12.3%)	.15‡
Facility	301	107 (35.5%)	155 (51.5%)	15 (5.0%)	24 (8.0%)	
Low Birthweight§, n (%)	157	68 (43.3%)	80 (51.0%)	2 (1.3%)	7 (4.5%)	**.022**†
Underweight§, n (%)	232	95 (40.9%)	116 (50.0%)	9 (3.9%)	12 (5.2%)	**<.001**†
Stunted§, n (%)	168	75 (44.6%)	80 (47.6%)	6 (3.6%)	7 (4.2%)	**<.001**†
Wasted§, n (%)	144	47 (32.6%)	75 (52.1%)	10 (6.9%)	12 (8.3%)	.20†
Any malnutrition§, n (%)	288	109 (37.8%)	146 (50.7%)	15 (5.2%)	18 (6.2%)	**<.001**‡
Infectious disease deaths§, n (%)	226	80 (35.4%)	111 (49.1%)	14 (6.2%)	21 (9.3%)	.81†
Lower Respiratory Infections, Sepsis, and Diarrheal Diseases§, n (%)	175	65 (37.1%)	85 (48.6%)	8 (4.6%)	17 (9.7%)	.42†

Weight-for-age (WAZ), length/height-for-age (LAZ), and weight-for-length/height (WLZ) z-scores <2 SD was used to define underweight, stunted, and wasted, respectively. Sample decrease within the underweight category due to 3 cases missing weight, 1 case missing age in days, and 1 case with WAZ outside of defined limits. Sample decrease within the stunted category due to 20 cases missing z-score due to lengths under 45 cm and 5 cases with LAZ outside of defined limits (refer to methods section). Sample decrease within the wasting category due to 117 cases missing z-score due to lengths under 45 cm, 3 cases with missing weight, 3 cases outside of the defined upper limit, and 1 case with outside the measurement limits for raw length captured by the World Health Organization growth standards (WHO-GS). Differences in sample size for wasting and stunting exist because LAZ is estimable based on length according to completed age and sex, whereas wasting is a measure of weight by length, details on the WHO-GS can be referenced elsewhere.^41^

Any malnutrition was defined WAZ, LAZ, or WLZ < -2 SD. Sample size drop in any malnutrition based on anthropometry at death due to 1 measurement outside of the WHO-GS bounds, and 7 cases with z-scores outside of the limits captured by the WHO-GS.

All Infectious diseases deaths included: diarrheal diseases, congenital infections, HIV, lower respiratory infections, malaria, measles, meningitis/encephalitis, neonatal sepsis/sepsis, other infections, rabies, syphilis, tuberculosis, or upper respiratory infections in the causal chain. Sub-analyses were conducted to investigate the association between vitamin A deficiency and infectious causes of death with an established relationship with vitamin A. These conditions included: lower respiratory infections, neonatal sepsis/sepsis, and diarrheal diseases.

∗ Vitamin A status was defined using cutoffs of <0.1 μmol/g, ≥0.1 to <0.7 μmol/g, ≥0.7 to <1.0 μmol/g, and ≥1.0 μmol/g to indicate VAD, adequate VA status, high VA, and hypervitaminosis, respectively.^1^

† Pearson's Chi-squared tests examining frequency distributions of demographic characteristics by Vitamin A Status.

‡ n (%).

§ Birthweight was abstracted from maternal or child clinical records. For deaths and stillbirths in the first 24 hours, if clinical abstraction data was missing, birthweight was assumed to be the MITS weight. Low birthweight was defined as <2500 g. Eighty-seven cases missing clinical abstraction data from maternal and child clinical records; 1 stillbirth or death in the first 24 hours was missing both clinical abstraction weight and MITS weight.

Table III summarizes the most frequent infectious and noninfectious CoD by age. CHAMPS deaths may have multiple conditions within the causal pathway (eg, immediate, underlying, and antecedent); therefore, the number of CoD could exceed the numbers of deaths in our sample (sum would therefore be greater than 100%). Overall, 226 deaths (61.8%) were attributed to an infectious disease. The top 3 infectious CoD overall included sepsis (31.7%, 116/366), LRI s (29.2%, 107/366), and malaria (10.9%, 40/366). The top 3 noninfectious CoD overall included neonatal preterm complications (27.9%, 102/366), perinatal asphyxia/hypoxia (20.2%, 74/366), and congenital birth defects (11.2%, 41/366).

**Table III:** Top infectious and noninfectious causes of death by age group among children under 5 years (n = 366) with analyzed liver specimens from CHAMPS Kenya and South Africa sites

	Overall n = 366	Stillbirths or death in the first 24 hours n = 97	Neonates (1 to 27 days) n = 98	Infants and children (28 days to < 60 months) n = 171
Infectious diseases	n (%)∗			
Sepsis	116 (31.7)	5 (5.2)	51 (52.0)	60 (35.1)
Lower respiratory infections	107 (29.2)	0 (0)	34 (34.7)	73 (42.7)
Malaria	40 (10.9)	0 (0)	0 (0)	40 (23.4)
Meningitis/Encephalitis	32 (8.7)	1 (1.0)	21 (21.4)	10 (5.8)
HIV	22 (6.0)	0 (0)	0 (0)	22 (12.9)
Diarrheal diseases	21 (5.7)	0 (0)	0 (0)	21 (12.3)
Congenital Infections	18 (4.9)	2 (2.1)	12 (12.2)	4 (2.3)
Other infections	11 (3.0)	0 (0)	1 (1.0)	10 (5.8)
Tuberculosis	2 (0.5)	0 (0)	0 (0)	2 (1.2)
Syphilis	1 (0.3)	0 (0)	0 (0)	1 (0.6)
Noninfectious Diseases				
Neonatal preterm birth complications	102 (27.9)	22 (22.7)	66 (67.3)	14 (8.2)
Perinatal asphyxia/hypoxia	74 (20.2)	67 (69.1)	6 (6.1)	1 (0.6)
Congenital birth defects	41 (11.2)	4 (4.1)	13 (13.3)	24 (14.0)
Malnutrition	36 (9.8)	0 (0)	0 (0)	36 (21.1)
Other neonatal disorders	33 (9.0)	5 (5.2)	27 (27.6)	1 (0.6)
Other respiratory disease	30 (8.2)	2 (2.1)	5 (5.1)	23 (13.5)
Injury	12 (3.3)	0 (0)	0 (0)	12 (7.0)
Neonatal aspiration syndromes	11 (3.0)	8 (8.2)	3 (3.1)	0 (0)
Neonatal encephalopathy	9 (2.5)	5 (5.2)	4 (4.1)	0 (0)
Undetermined	7 (1.9)	3 (3.1)	0 (0)	4 (2.3)
Kidney Disease	5 (1.4)	0 (0)	5 (5.1)	0 (0)
Other neurological disorders	5 (1.4)	1 (1.0)	1 (1.0)	3 (1.8)
Other	4 (1.1)	0 (0)	1 (1.0)	3 (1.8)
Liver disease	3 (0.8)	0 (0)	1 (1.0)	2 (1.2)
Poisoning	3 (0.8)	0 (0)	0 (0)	3 (1.8)
Sickle cell disorders	3 (0.8)	0 (0)	0 (0)	3 (1.8)
Other disorders of fluid, electrolyte and acid-base balance	2 (0.5)	0 (0)	1 (1.0)	1 (0.6)
Other endocrine, metabolic, blood, and immune disorders	2 (0.5)	0 (0)	0 (0)	2 (1.2)
Anemias	1 (0.3)	0 (0)	0 (0)	1 (0.6)
Cancer	1 (0.3)	0 (0)	0 (0)	1 (0.6)
Epilepsy	1 (0.3)	0 (0)	0 (0)	1 (0.6)
Heart diseases	1 (0.3)	0 (0)	0 (0)	1 (0.6)
Other gastrointestinal disease	1 (0.3)	0 (0)	0 (0)	1 (0.6)
Other immunodeficiencies	1 (0.3)	0 (0)	0 (0)	1 (0.6)
Other skin and subcutaneous diseases	1 (0.3)	0 (0)	0 (0)	1 (0.6)
Paralytic ileus and intestinal obstruction	1 (0.3)	0 (0)	0 (0)	1 (0.6)
Placental complications	1 (0.3)	0 (0)	1 (1.0)	0 (0)
Umbilical cord complications	1 (0.3)	1 (1.0)	0 (0)	0 (0)

∗ Deaths may have multiple conditions in the causal pathway (eg, underlying, immediate, and antecedent), therefore the number of causes of death could exceed the number of deaths (and the sum would be >100%).

**Figure 2:**
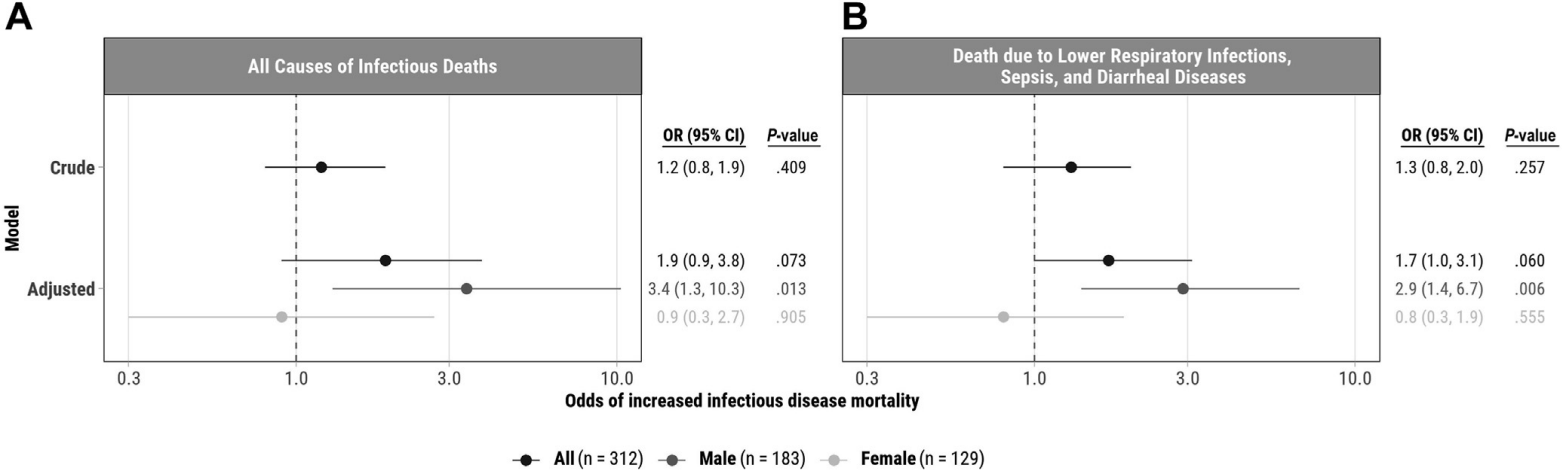
Crude and adjusted models of the association between vitamin A deficiency and odds of dying of infectious causes of death among children under 5 years with analyzed liver specimens from CHAMPS Kenya and South Africa Sites, n = 312 children. The crude model depicts the unadjusted relationship between vitamin A deficiency and infectious causes of death. Adjusted models depict the relationship between vitamin A deficiency and infectious causes of death controlling for site, age, and sex. Box A. All causes of infectious deaths defined as deaths due to diarrheal diseases, congenital infections, HIV, lower respiratory infection, malaria, measles, meningitis/encephalitis, sepsis, other infections, rabies, syphilis, tuberculosis, or upper respiratory infection in the causal chain. Box B. Sub-analysis of the most common causes of infectious disease mortality which have an established relationship with vitamin A deficiency. These conditions included lower respiratory infection, sepsis, and diarrheal diseases.

Crude models examining the association between VAD and infectious CoD were not significant (Figure 2). When adjusting for site, sex, and age, there was no significant association between VAD and increased infectious CoDs (OR 1.9, 95%CI 0.9, 3.8, *P* = .073). We did, however, note heterogeneity by sex (*P* = .09), and thus performed stratified analysis. Boys with VAD, but not girls, had a significantly increased risk of mortality due to infectious CoDs (OR_boys_ 3.4, 95%CI 1.3, 10.3, *P* = .013; OR_girls_ 0.9, 95%CI 0.3, 2.7, *P* = .905). LBW and indicators of malnutrition at death were either nonsignificant predictors of cause of mortality or did not improve model fit and thus were not included in final models.

Sub-analysis of specific infectious CoDs (LRIs, sepsis, and diarrheal diseases) did not reveal a significant association between VAD and increased mortality due to LRIs, sepsis, and diarrhea (OR: 1.7, 95% CI 1.0, 3.1, *P* = .06), but showed significant effect modification by sex (*P* = .02). Similar to the primary outcome of all infectious CoD, boys, but not girls, had a significantly increased risk of death due to LRIs, sepsis, or diarrheal CoDs in the final adjusted model (OR_boys_ 2.9, 95% CI 1.4, 6.7, *P* = .006; OR_girls_ 0.8, 95% CI 0.3, 1.9, *P* = .555). Final models were stratified by age group, and VAD was associated with all causes of infectious deaths among stillbirths or deaths in the first 24 hours (OR 8.8, 95% CI 1.3, 176.0) and infants and children (OR 3.8, 95% CI 0.97-25.6), but not among neonates (OR 1.0 95% CI 0.4, 2.4). Sample sizes were too low to assess the association of VAD and mortality due to LRIs, sepsis, and diarrhea stratified by age.

## Discussion

This large, multisite study of reference-standard postmortem liver VA concentrations in children under 5 years of age suggests coexistence of VAD and excess in Kenya and South Africa, both settings with ongoing public health interventions designed to prevent VAD (eg, VAS, food fortification). Furthermore, we found increased odds of dying of all infectious CoD as well as LRIs, sepsis, and diarrheal diseases among boys with VAD, but not among girls.

WHO defines VAD as a severe public health problem if the national prevalence of serum retinol <0.7 μmol/L in PSC exceeds 20%.^1^ According to the latest national nutrition survey in South Africa in 2012, the prevalence of VAD among PSC was 44%.^43^ The 2011 Kenya National Micronutrient Survey found 9.2% of PSC with VAD, but 52.6% of PSC had marginal VAD (serum retinol: 0.7-1.05 μmol/L) and thus were at risk of becoming deficient.^44^ The Micronutrient Forum recommends that countries complete a micronutrient survey every 5 years to track changes in status and to inform national nutrition programs.^45^

Our study's key strength lies in the use of reference-standard TLVAC, which can be used to classify individuals across the VA continuum, from deficiency through excess.^1^^,^^4^^,^^14^, ^15^, ^16^, ^17^, ^18^ Commonly used biomarkers of VA status, including serum retinol and RBP concentrations, are subject to limitations in both clinical and public health settings. Serum retinol is homeostatically controlled and does not decrease until liver reserves of VA are depleted. Additionally, both retinol and RBP decrease in the presence of inflammation or infection, which can contribute to overestimation of VAD.^46^ Both RBP and serum retinol may miss excess or toxic levels of VA, which may be helpful in calibrating VA programs. The utility of assessing TLVAC within mortality surveillance as a more reliable tool for monitoring population VA status warrants additional research.

The observed coexistence of VAD and VA excess may be present due to various factors including dietary intake, poverty, food insecurity,^47^^,^^48^ cultural perceptions around food,^49^ and access to health services. Both Kenya and South Africa have mandatory staple food VA fortification programs (vegetable fats and oil in Kenya and wheat and maize flour in South Africa) as well as universal biannual VAS among 6-59 month olds^33^^,^^50^; however, VAS coverage varies between 30-80% in Kenya and 30-40% in South Africa. Incomplete and duplicative VAS coverage may explain the coexistence of deficiency and excess in the same population.^48^^,^^51^ In 1 area of South Africa, the number of capsules received through VAS was directly related to increasing liver reserves.^52^ Across five African countries, both deficiency and excessive stores of VA have been documented.^53^

In Kenya, the latest Demographic and Health Survey data suggest that 3 in 4 children under 2 years of age receive VA-rich food sources as well as VAS, which may help contextualize the adequate to high VA levels in nearly two-thirds of children.^54^ In South Africa, traditional beliefs around appropriate foods during pregnancy and early life may contribute to both deficiency and excess VA. For example, in the Northern Cape province of South Africa where sheep farming is a main agricultural activity, 41-70% of children 12-23 months of age consume liver, an exceptionally high source of preformed VA^32^^,^^55^; whereas limited consumption of orange color, VA rich plant-based foods and meat occurs during pregnancy for fear of adverse or undesirable traits in the baby, which may contribute to VAD.^49^ Therefore, the coexistence of VAD and excess in Kenya and South Africa may be attributed to a complex interplay of poverty, food insecurity, cultural food practices and beliefs, and overlapping VA interventions.

VADhas a significant impact on U5M, and studies have shown that VA deficient children are more susceptible to infections, such as measles, diarrhea, and respiratory diseases^6^, ^7^, ^8^, ^9^ and are therefore at greater risk of U5M. Inadequate intake of VA can weaken the immune system, making children more susceptible to illnesses that lead to death. The complex interplay between VA, infection, and mortality^56^ may elucidate the mechanisms by which children with VAD in our study were found to have elevated odds, although not significant, of dying from an infectious disease. Our study also suggests that the effects of VAD on U5M may vary by sex. This finding complements existing literature that suggests that boys have a higher U5M than girls in many low and middle-income countries, and that VASin early infancy may have more beneficial effects on mortality for males than for females.^57^ Further high-powered research is needed to better understand sex differences in requirements for VA, factors associated with sex inequality in U5M, and the impacts of targeted interventions and management of selective threats to male survival including prematurity and early childhood infection.^58^ Future studies may consider replication of this study across all CHAMPS sites.

This study has several strengths. First, this study is innovative in its use of postmortem liver samples to conduct reference-standard VA assessment through MITS. The MITS technique is a low-cost, nondisfiguring, needle-based approach to acquire postmortem samples. Previous studies have shown that MITS samples are highly correlated with complete diagnostic autopsies.^21^ Additionally, previous research on the acceptability and feasibility of this approach with community participants found that not only did families consent to MITS at higher than anticipated rates (75%), but an overwhelming majority of parents expressed relief and gratitude for being provided information about why their child died.^20^^,^^21^^,^^25^^,^^29^ Second, this is a large and recent assessment of TLVACs using postmortem livers. While previous studies have utilized postmortem liver specimens for reference-standard VA assessment, these early studies often lacked sufficient sample size, failed to have representation from the first 5 years of life, and lacked data from countries with high U5M rates.^15^^,^^16^^,^^59^ Third, the timeliness of specimen collection is a key strength of our study. Previous literature has shown that postmortem liver is viable up to 108 hours after death. Our samples were collected within 72 hours after death.

Our study has limitations. First, the cross-sectional nature of our data does not allow inference of causality. While certain infections themselves may cause VADdue to increased nutrient utilization or losses, our analyses were informed by the presumed causality of VAD and infectious mortality, as confirmed by both biology and prior randomized clinical trials. Further, the association between VAD and infectious CoD was consistent across age groups, even in stillbirths or deaths in the first 24 hours who likely had in utero VAD due to poor VA supply in pregnancy rather than acquiring it from an infection. Second, we lack data on dietary patterns, fortification coverage, and history of VAS that may be helpful to contextualize the coexistence of deficiency and excess in our sample. Third, we cannot control the variability of antemortem data; eg, data on VAS from child clinical abstraction was either missing or unknown for most of our samples. Further, due to lack of availability of data we were not able to explore the impact of poverty and other sociodemographic factors in our models. Fourth, the size of the liver sample was limited and duplicate analysis could not be performed, which would have been the case for low extraction efficiencies. Degradation of VA in liver also could have impacted samples with delayed collection after death, as could potential differences in VA from liver samples collected from different lobes. Finally, CHAMPS is an ongoing surveillance system and is refined through lessons learned through network sharing and in-person process monitoring. This means that results and conclusions using these data can change over time.

A unique challenge facing policy makers will be determining the appropriate balance of VA interventions, as deficiency and excess may coexist in the same population or differ among various subgroups. There is a continued need for routine population-based assessment of micronutrient status that includes dietary data, reliable biological indicators of VA status including TLVAC as a data source,^60^^,^^61^ and consumption patterns of industrially fortified VA staple foods. Together, these data may be used to inform future changes to universal VAS strategies, including a transition from universal VAS to targeted strategies in certain countries.

## Data Statement

Data sharing statement available at www.jpeds.com.

## Declaration of Competing Interest

This work was funded by grant OPP1126780 from the 10.13039/100000865Bill & Melinda Gates Foundation and a seed grant from the 10.13039/100006090Global Health Institute at 10.13039/100007015University of Wisconsin-Madison.

The funders participated in discussions of study design and data collection. They did not participate in the conduct of the study; the management, analysis, or interpretation of the data; preparation, review, or approval of the manuscript; or decision to submit the manuscript for publication.

The authors declare no conflicts of interest.

## Supplementary Data

STROBE_checklist

Data Statement
