# Supplementary material for: Hepatic Vitamin A Concentrations and Association with Infectious Causes of Child Death
Source: J Pediatr. 2024 Feb;265:113816. doi: 10.1016/j.jpeds.2023.113816 (PMC10869935; doi:10.1016/j.jpeds.2023.113816)
Supplement: Data Statement [file mmc2.docx]

**Data Sharing:** Data described in the manuscript, code book, and analytic code will be made available upon request.
